# An analysis of the kinetics of molecular response during the first trimester of treatment with nilotinib in newly diagnosed chronic myeloid leukemia patients in chronic phase

**DOI:** 10.1007/s00432-017-2445-z

**Published:** 2017-05-27

**Authors:** Juan Luis Steegmann, Dolors Colomer, Maria-Teresa Gómez-Casares, Valentín García-Gutiérrez, Guillermo Ortí, Angel Ramírez-Payer, Eduardo Olavarria, Ferrán Vall-llovera, Pilar Giraldo, Eulogio Conde, Rolando Vallansot, Jose Luis López-Lorenzo, Luis Palomera, Alberto Álvarez-Larrán, Venancio Conesa, Guiomar Bautista, Laura Casas, Frank Giles, Andreas Hochhaus, Luis Felipe Casado-Montero

**Affiliations:** 1 Hematology Service, Advanced Therapies in Oncohematology, IIS-IP, Hospital de la Princesa, Madrid, Spain; 2 Unitat d’ Hematopatologia, Hospital Clinic, Barcelona, Spain; 3 Laboratorio de Hematología, Biología Molecular, Hospital Universitario de Gran Canaria Dr.Negrín, Las Palmas, Spain; 4 Servicio de Hematología, Hospital Universitario Ramón y Cajal, IRYCIS, Madrid, Spain; 5 Hematology Department, Hospital Universitari Vall d´Hebron, VHIO, Barcelona, Spain; 6 Servicio de Hematología, Hospital Universitario Central de Asturias, Oviedo, Spain; 7 Hematology Department, Imperial College Healthcare NHS Trust, London, UK; 8 Servicio de Hematología, Hospital Universitari Mútua Terrassa, Terrassa, Spain; 9 Instituto Investigación Sanitaria Aragón (IIS Aragon), CIBERER, Zaragoza, Spain; 10 Hematologia, Universidad de Cantabria, Santander, Spain; 11 Servei d´Hematologia, ICO-Tarragona, Hospital Universitari Joan XXIII, Tarragona, Spain; 12 Hematologia, Clínica de la Concepción, Madrid, Spain; 13 Hospital Clínico Universitario Lozano Blesa, Instituto Investigación (ISS) Aragón, Zaragoza, Spain; 14 Hematology Department, Hospital del Mar-IMIM, Barcelona, Spain; 15 Hematología, Departament de Salut d’Elx, Hospital General, Elche, Spain; 16 Hematología, Hospital Puerta de Hierro, Madrid, Spain; 17 Statistics Department, Dynamic Solutions, SL, Barcelona, Spain; 18 Division of Hematology Oncology, Northwestern University Feinberg School of Medicine, Chicago, USA; 19 Klinik für Innere Medizin II. Hämatologie/Onkologie, Universitätsklinikum Jena, Jena, Germany; 20 Servicio de Hematología y Hemoterapia, Hospital Virgen de la Salud, Toledo, Spain

**Keywords:** Chronic myeloid leukemia, Nilotinib, ENEST1st

## Abstract

**Purpose:**

This study was aimed to analyze the association of very early molecular response to nilotinib with the achievement of deep molecular response (MR4) at 18 months. We hypothesized that the BCR-ABL1 levels during the first 3 months of therapy, and the kinetics of their descent in this period, could be predictive of deep molecular response thereafter.

**Methods:**

This substudy of the ENEST1st trial included 60 patients with chronic myeloid leukemia in chronic phase treated with front-line nilotinib, and BCR-ABL1IS levels were measured using GUS as the control gene. The analysis included seven time points during the first trimester of treatment (baseline and fortnightly thereafter).

**Results:**

The rates of MMR at 12 months, and of MR4 at 18 months (primary variable of the study), were 70 and 41%, respectively, similar to those obtained in the core study. BCR-ABL1IS ≤10% was achieved at 1, 1.5, 2 and 3 months in 50, 70, 83 and 93% of the patients, respectively. The observed shape of the BCR-ABL1IS descent was biphasic, with a faster slope during the first trimester and a median halving time (HT) of 11 days, the shortest reported in the literature. An HT ≤13 days was predictive of MMR at 12 months and MR4 at 18 months.

**Conclusions:**

The association of a shorter HT with response provides a rationale for exploring very early kinetics patterns in all patients treated with potent TKIs such as nilotinib.

## Introduction

Nilotinib (Tasigna, Novartis Pharmaceuticals Corporation, East Hanover, NJ, USA) is a BCR-ABL1 tyrosine kinase inhibitor (TKI) approved for the treatment of newly diagnosed Philadelphia chromosome-positive (Ph+) chronic myeloid leukemia in chronic phase (CML-CP) (Corporation 2015). With 5 years of follow-up of the ENESTnd study, nilotinib showed improved efficacy over imatinib in the frontline setting for patients with CML-CP, including earlier and deeper molecular responses and a low rate of progression to AP/BC (Hochhaus et al. 2016b). Besides, nilotinib led to higher rates of *BCR*-*ABL1* ^*IS*^ ≤10% and *BCR*-*ABL1* ^*IS*^ ≤1% at 3 months than imatinib.(Saglio et al. 2010) ENEST1st was a phase 3b, multicenter, single-arm open-label study evaluating the efficacy and safety of nilotinib in a large population of patients with newly diagnosed Ph+ CML-CP, with an emphasis on deep molecular response. The primary end point was MR^4^ (*BCR*-*ABL1* ^*IS*^ ≤0.01%) at 18 months. In patients treated with nilotinib upfront, the rate of MR^4^ at 18 months was 38.3%, and the proportion of patients of the landmark population having *BCR*-*ABL1* ^*IS*^ ≤10% by 3 months was 97%(Hochhaus et al. 2016b). Patients with a ratio ≤10% at 3 months achieved the highest rates of response at later time points, whereas no patient with a ratio >10% at 3 months achieved MR^4^ by 24 months.

Data of the kinetics during the first trimester are available in patients treated with frontline imatinib, nilotinib, and dasatinib, but there is a great heterogeneity in the chosen time points. Most studies have used *BCR*-*ABL1* ^*IS*^ levels at diagnosis (Branford et al. 2014; Hanfstein et al. 2014; Huet et al. 2014; Michor et al. 2005; Olshen et al. 2014; Tang et al. 2011) as baseline parameters, whereas only one study has used ratios obtained just before treatment as baseline levels (Iriyama et al. 2015). In addition, most studies have used only the 3-month milestone to calculate the kinetics, and only one has used data at the first month as intermediate measurement (Iriyama et al. 2015). Given the scarcity and heterogeneity of the data on the kinetics of the descent of *BCR*-*ABL1* ^*IS*^ in patients treated with nilotinib upfront, the purpose of our study was to analyze the kinetics of the transcript’s descent using seven time points during the first trimester and to establish if this kinetics has a predictive value on subsequent response.

Our hypothesis was that deep molecular response to nilotinib would be associated with the values of *BCR*-*ABL1* ^*IS*^ during the first 3 months of therapy and the kinetics of their descent during this period.

## Patients and methods

ENEST1st was registered in the EU Clinical Trials Registry (2009-017775-19) and at clinicaltrials.gov (NCT01061177). Adults (aged ≥18 years) with newly diagnosed (≤6 months), cytogenetically confirmed Ph+ CML-CP or Ph-BCR-ABL1+ CML-CP were eligible for enrollment. In this particular substudy, imatinib was not allowed prior to nilotinib, but hydroxyurea (HU) was permitted (≤6 months). This study was conducted in accordance with the International Conference on Harmonization Harmonized Tripartite Guidelines for Good Clinical Practice, the Declaration of Helsinki, and applicable local regulations. Eligible patients were only included in the study after providing written consent and in accordance with local laws/regulations. The protocol and informed consent forms were reviewed and approved by an institutional review board, independent ethics committee, or research ethics board prior to study start at each participating institution. All enrolled patients received nilotinib 300 mg twice daily for up to 24 months. Dose escalation of nilotinib was not allowed. Nilotinib dose reduction was required for patients with grade 3/4 hematologic adverse events (AEs) concerning white blood cells and platelets (not concerning hemoglobin level), or grade 2, 3, or 4 non-hematologic AEs. The primary objective was to find a level of *BCR*-*ABL1* ^*IS*^ within the first 3 months that has prognostic value on the primary variable of the core study, i.e., MR^4^ (*BCR*-*ABL1* ^*IS*^ ≤0.01%) at 18 months. The secondary objectives of this substudy were to evaluate the kinetics of the reduction of the *BCR*-*ABL1* ^*IS*^, and to study a potential association of kinetic variables and the attainment of MR^4^ at 18 months. The classification of response was made according to standard definitions (Cross et al. 2012, 2015). Major molecular response (MMR), defined as a *BCR*-*ABL1* ^*IS*^ ≤0.1%, MR^4^ (defined as detectable *BCR*-*ABL1* ^*IS*^ ≤0.01% or undetectable *BCR*-*ABL1* in samples with ≥24 000 *GUS* transcripts), and MR^4.5^ (defined as detectable *BCR*-*ABL1*1^IS^ ≤0.0032% or undetectable *BCR*-*ABL1* in samples with ≥77 000 *GUS* transcripts).

### Assessments

The BCR-ABL1 transcript type was determined by multiplex PCR at baseline (Cross et al. 1994). Only patients with typical transcripts (e13a2 and/or e14a2) were included. Molecular responses were assessed at baseline (i.e., just before the first dose of nilotinib was given), and then every 15 days (±2) during the first 3 months and on months 6, 9, 12, 18 and 24 during study treatment. Samples were analyzed using real-time quantitative polymerase chain reaction (RQ-PCR) at a designated EUTOS reference laboratory. Samples were analyzed using *GUS* as control genes. AEs were assessed according to the Common Terminology Criteria for Adverse Events (CTAE) version 4.0.

## Statistical analyses

Summary data for demographic variables and baseline characteristics were determined for the intent-to-treat (ITT) patient population, which included all patients who received ≥1 dose of study treatment. Efficacy analyses were performed on the ITT population. Response rates are presented as the percentage of patients with the response at the specified time point.

### Independent variables

Independent variables at diagnosis included in the analysis were those of Sokal, Hasford and EUTOS scores. Besides, the following independent variables were obtained at baseline (i.e., just before nilotinib initiation) and included in the analysis: *BCR*-*ABL1* ^IS^, spleen size, leukocyte number, percentages of basophils and immature granulocytes. Differential counts were centrally assessed. Independent variables during the treatment were: *BCR*-*ABL1* ^*IS*^ obtained at 15, 30, 45, 60, 75 and 90 days of treatment, the ratios between them and the baseline ratios and halving times (HT). Descriptive statistics were used for independent and dependent variables. Correlations between numerical variables were calculated by the Pearson or Spearman’s correlation coefficient, depending of the distribution of the variables.

### Analysis of the kinetics of the descent of the *BCR*-*ABL1*^*IS*^

*BCR*-*ABL1* ^*I*^ measurements were done every 15 days in the first 3 months of therapy. As a coarse estimation of kinetics, we calculated the ratios between the *BCR*-*ABL1* ^*IS*^ values obtained during the first trimester. For example, *BCR*-*ABL1* ^*IS*^ at 3 m/*BCR*-*ABL1* ^*IS*^ at baseline, *BCR*-*ABL1* ^*IS*^ at 3 m/*BCR*-*ABL1* ^*IS*^ at day 15 and so forth. Logarithmic transformations in base 10 of the *BCR*-*ABL1* ^*IS*^ *levels* were made in each visit. After that, slopes per day were calculated since baseline until the third month and from this month to the 18 month, following the method by Michor et al. (2005). To determine if the diminution of the ratio fits a constant logarithm, the Pearson’s correlation coefficient between the time and the variable log10 (*BCR*-*ABL1* ^*IS*^) was calculated. We accepted that the reduction was constant logarithmic in those patients who had a correlation coefficient near −1 and with a *p* < 0.05. With this method, we selected 49 patients out of 57. HT was calculated using the method of logarithmic transformation (Branford et al. 2014) for the following time points: day 15, day 30, day 45, months 2 and 3. It was calculated as follows: HT = −ln (2)/*k*, where *k* = [ln(ratio tx) − ln(baseline ratio)/number of days between the measurements]. the *k* coefficient measures the velocity of diminution in this given interval of time. When there was no reduction of the ratio, the HT was negative. These values were excluded from the analysis. Molecular response was analyzed at every time point, according to international guidelines (Cross et al. 2012). To analyze the impact of the independent variables on subsequent variables of molecular response, we performed a logistic regression analysis independently for each time point, using the “enter” method. The analysis was done by two methods. First, by entering all the independent variables and, second, entering only the variables at diagnosis and those obtained during the first 3 months. Odd ratios (OR) with correspondent *p* values and confidence intervals (CI) were estimated. If several variables achieved statistic significance, a multivariate logistic regression was used. To find a threshold that could predict subsequent responses, a receiver operating curve analysis (ROC) was performed. The safety population was identical to the ITT population.

## Results

### Patients and treatment exposure

In total, 61 patients were included in this substudy. The demographic characteristics are depicted in Table 1. One patient died just after the baseline visit, before receiving the first dose of nilotinib and was excluded for analysis. Of patients in the ITT population, 66.7% (*n* = 40) were male. The median age was 52 years (range 19–81 years). The median time since diagnosis was 0.6 months (range 0.03–4.4), and 41.7% (*n* = 25) of patients had received HU as prior treatment for CML, with a median duration of 15 days (1–120). The baseline ratios were not significantly different between patients with or without previous HU (39.6 ± 32 vs. 56.3 ± 35, *p* = 0.078). No other TKI was allowed prior to nilotinib. The majority of patients (96.7%; *n* = 58) had low EUTOS risk scores; Sokal risk scores were low, intermediate and high in 60% (*n* = 36), 28.3% (*n* = 17), and 11.7% (*n* = 7) of patients, respectively. The corresponding figures for Hasford scores were 52% (*n* = 31), 42% (*n* = 25) and 5% (*n* = 3), respectively. Fifty patients (83.3%) completed ≥18 months of study treatment. Ten patients (16.7%) discontinued study treatment before 18 months, and the reason for discontinuation was AEs or laboratory abnormalities (Table 2). The median duration of nilotinib exposure was 23.8 months (range 0.6–29.2). Dose changes or interruptions occurred in 36.7% (*n* = 22) of patients and were most commonly due to AEs or laboratory abnormalities (89.6%) or dosing error (7.8%). The median dose intensity was 593 mg/day (range 133–597).

**Table 1:** Demographic characteristics

At diagnosis			
Sex (M/F)	40/20	66.7%/33.3%	
Age (years)	51.8	19.4–80.6	
Spleen (cm)	0	0–22	
Sokal	36/17/7	60%/28.3%/11.7%	
Hasford	31/25/3	52.5%/42.4%/5.1%	
EUTOS	58/2	96.7%/3.3%	
At baseline	Median	Range	Mean ± SD
Months diagnosis: nilotinib	0.64	0.03–4.4	0.93 ± 0.91
Leukocytes	29.1	3.2–245.1	52.8 ± 52.5
Basophils	4	0–21.5	4.8 ± 4.1

**Table 2:** Reasons for discontinuation

NID	Time off-study	Cause
3414-2	Month 14	Hypophosphatemia
3442-2	Month 12	Creatinine elevation
3438-1	Month 10	Acute myocardial infarction
3417-1	Month 9	Creatinine elevation
3410-3	Month 9	CK elevation
3438-4	Month 9	Neutropenia
3412-1	Month 6	Acute myocardial infarction
3410-2	Month 6	GGT elevation
3423-1	Month 5	GOT/GPT elevation
3403-2	D15	Lipase elevation

### Response rates

Table 3 depicts the value of *BCR*-*ABL1* ^*IS*^ using GUS as reference gene at different time points, including baseline. MR^4^ at 18 months was obtained in 29 patients (48.3%). The rates of MMR at 12 and 18 months were 70% (*n* = 42) and 68.3% (*n* = 41), respectively. A ratio ≤10% at 3 months was achieved in 93.3% of the patients. The correspondent percentages for 1, 1.5 and 2 months were 50, 70 and 83%, respectively. Three-quarters of the patients obtained a ratio ≤1% at 3 months, and 38% obtained MMR at this time point (Table 4).

**Table 3:** *BCR*-*ABL1/GUS* ratios (IS)

% BCR-ABL/GUS	*N*	Mean	SD	Median	Min	Max
Baseline	57	33.53	33.66	23.52	3.37	148.20
Day 15	56	22.14	21.19	15.17	0.02	98.07
1 Month	56	13.03	13.19	8.49	0.71	60.45
Day 45	57	8.99	15.97	3.95	0.05	107.91
2 Months	56	4.18	7.98	1.34	0.02	32.95
Day 75	51	1.118	2.03	0.25	0.02	10.16
3 Months	56	0.72	1.48	0.14	0.0004	7.80
6 Months	54	0.45	1.39	0.02	0.00	6.77
12 Months	50	0.09	0.29	0.004	0.00	1.51
18 Months	46	0.05	0.11	0.01	0.00	0.46

**Table 4:** Molecular response with GUS as the control gene (ITT)

	Ratio ≤10%	Ratio ≤1%	MMR ≤0.1%	MR4 ≤0.01%	MR4.5 ≤0.0032%
BCR-ABL/GUS					
1 M	30/60 (50%)	2/60 (3.3%)	0/60 (0%)	0/60 (0%)	0/60 (0%)
1.5 M	42/60 (70%)	9/60 (15%)	1/60 (1.7%)	0/60 (0%)	0/60 (0%)
2 M	50/60 (83.3%)	25/60 (41.7%)	2/60 (3.3%)	0/60 (0%)	0/60 (0%)
3 M	56/60 (93.3%)	45/60 (75%)	23/60 (38.3%)	9/60 (15%)	5/60 (8.3%)
6 M	54/60 (90%)	50/60 (83.3%)	35/60 (58.3%)	19/60 (31.7%)	10/60 (16.7%)
12 M	50/60 (83.3%)	48/60 (80%)	42/60 (70%)	31/60 (51.7%)	20/60 (33.3%)
18 M	46/60 (76.7%)	46/60 (76.7%)	41/60 (68.3%)	29/60 (48.3%)	11/60 (18.3%)

### Kinetics of the descent of the transcript

The curve of descent of *BCR*-*ABL1* values was biphasic, with a faster slope during the first trimester. The slope per day during the first trimester was −0.052. The corresponding figure for the period between 3 and 18 months was −0.003. As we had values available every 15 days, HT was calculated with values at every time point. When there was no reduction of the ratio, the HT was negative. This happened in 19 cases for the estimation made at 15 days, 9 cases at 1 month and 5 cases at 45 days. These cases were excluded of the analysis. The value of HT with measurements taken at 3 months showed a median HT of 11.1 days (range 6.3–29.4) (mean ± SD: 12.7 ± 5.3 days).

### Analysis of the variables influencing the response

Table 5 depicts the variables found significant in the univariate and multivariate analysis. For the purpose of this analysis, we used the value of HT with ratios taken at 3 months. Baseline *BCR*-*ABL1* values were not significantly associated with subsequent responses in univariate analysis. In multivariate analysis, high Sokal score remained significantly associated with lower probability of MMR at 18 months. Larger spleen size was linked with lower probability of MR^4^ at 18 months. The ratio at 3 months was associated with subsequent responses. Having a ratio ≤0.19 at 3 months was associated with a 75% probability of having an MR^4^ at 18 months.

**Table 5:** Analysis of molecular response using *BCR*-*ABL1*/GUS ratios

	Variables found significant in the univariate analysis	Variables found significant in multivariate analysis	Best model of ROC
MMR 12 m	Sokal (*p* = 0.06)	**Halving time** **OR: 0.35 (0.15–0.80)** ***p*** **=** **0.013**	
	**Ratio at 3** **m (** ***p*** **=** **0.023)**	**Ratio 3** **m/baseline ratio** **(** ***p*** **=** **0.025)**	
	**Ratio at 6** **m (** ***p*** **=** 0.013)*		
	**Ratio at 3** **m/baseline ratio (** ***p*** **<** **0.001)**		
	**Ratio at 3** **m/ratio d45 (** ***p*** **<** **0.002)**		
	**Ratio ≤1% at 3** **m (** ***p*** **=** **0.006)**		
	**MMR at 6** **m (** ***p*** **=** 0.001)*		
	**Halving time (** ***p*** **=** **0.001)**		
MMR 18 m	Sokal (*p* = 0.068)	**Sokal** **OR: 0.088 (0.008–0.935)** ***p*** **=** **0.044**	
	**Ratio at 3** **m (** ***p*** **=** **0.037)**		
	**Ratio at 12** **m (** ***p*** **=** 0.012)*		
	**Ratio at 3** **m/baseline ratio (** ***p*** **<** **0.023)**		
		**MMR at 3** **m** **OR: 22.1 (1.81–270)** ***p*** **=** **0.015**	
	**Ratio at 3** **m/ratio d45 (** ***p*** **=** **0.075)**		
	**Ratio ≤1% at 3** **m (** ***p*** **=** **0.038)**		
	**MMR at 3** **m (** ***p*** **=** **0.018)**		
	**MMR at 6** **m (** ***p*** **=** 0.006)*		
MR4 18 m	Spleen size (*p* = 0.051)	**Spleen size** **OR: 0.74 (0.57–0.96)** ***p*** **=** **0.027**	**Ratio at 3** **m** **AUC: 0.76** **Cutoff: 0.19** **PPV: 75% NPV: 75.9%**
	**Ratio at 3** **m (** ***p*** **=** **0.035)**		
	**Ratio at 6** **m (** ***p*** **=** 0.027)*		
	**Ratio at 12** **m (** ***p*** **=** 0.019)*		
		**MMR at 3** **m** **OR: 7.15 (1.77–28.7)** ***p*** **=** **0.006**	
	**Ratio at 3** **m/baseline ratio (** ***p*** **<** **0.004)**		
	**Ratio at 3** **m/ratio d45 (** ***p*** **=** **0.023)**		
	**Ratio ≤1% at 3** **m (** ***p*** **=** **0.044)**		
	**MMR at 3** **m (** ***p*** **=** **0.006)**		
	**MMR at 6** **m (** ***p*** **=** 0.00003)*		
	**Halving time (** ***p*** **=** **0.029)**		

Association between independent variables and subsequent responses

In bold case, variables found significant. Normal case, trend. Baseline ratio was not found to be significantly associated with response. For MMR 12 m: *p* = 0.874; MMR 18 m *p* = 0.578; MR4 18 m: *p* = 0.310. The value for HT was that obtained at 3 months

* Excluded from multivariate analysis

**Fig. 1:**
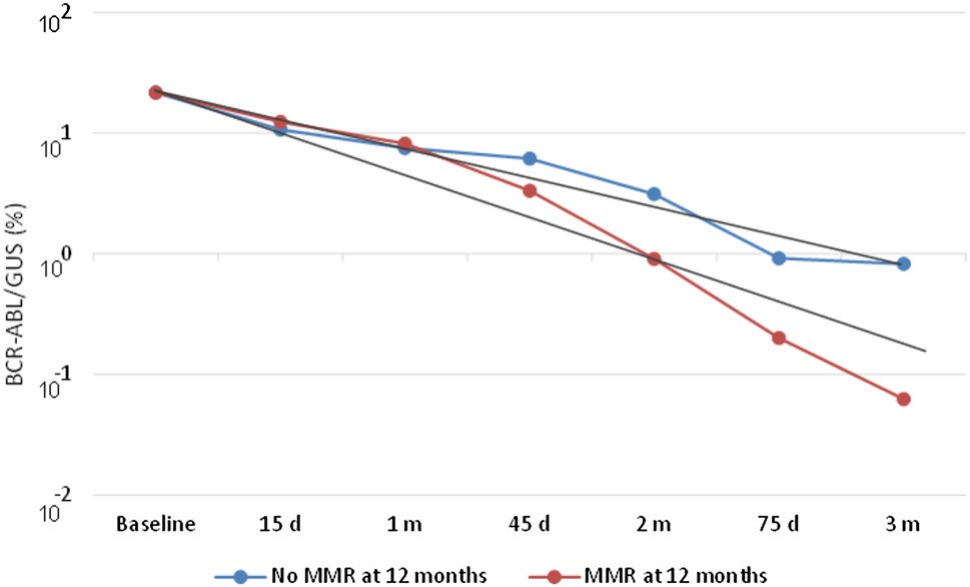
Kinetics of the BCR-ABL/GUS ratio with time in patients with and without MMR at 12 months. Slopes per day were significantly higher in patients having MMR at 12 months (Mean ± SD: −0.057 ± 0.01) than in those without MMR at 12 months (Mean ± SD: −0.032 ± 0.02). Halving time was significantly lower in those patients with MMR (11.8 ± 5.1 days) than in those with no MMR at 12 months (16.9 ± 4.6 days) (*p* = 0.001)

As depicted in Fig. 1, a faster kinetics was associated with response. In fact, the halving time was significantly lower in those patients with MMR (11.8 ± 5.1 days) than in patients with no MMR at 12 months (16.9 ± 4.6 days) (*p* = 0.001). Using ROC, we found that a value of 13 days had a sensitivity of 89%, specificity of 78%, PPV of 50% and NPV of 97%. OR: 29 (*p* = 0.003). This means that having an HT ≤13 days was associated with a 97% probability of having an MMR at 12 months. For MR^4^ at 18 months, HT was significantly lower in those patients with MR^4^ (11.6 days ± 4.8) than in patients with no MR^4^ (14.8 ± 5.7) (*p* = 0.029). Using ROC, we found that a value of 13 had a sensitivity of 58%, specificity of 81%, PPV of 69% and NPV of 72%. This means that having an HT ≤13 days was associated with a 72% probability of having an MR^4^ at 18 months. When entering HT in a multivariate analysis, HT and the ratio *BCR*-*ABL* ^*IS*^ 3 M/*BCR*-*ABL* ^*IS*^ baseline were the only two variables independently associated with MMR at 12 months, but HT was not independently associated with MR^4^ at 18 months.

### Safety

Most of the reasons for permanent discontinuation in our study were because of laboratory abnormalities. Two patients discontinued the study because of ischemic heart disease (acute myocardial infarction). There were no cases of disease progression or deaths in our series.

## Discussion

The proportion of patients in whom the primary end point, MR^4^ at 18 months, was achieved was 48%, similar to that obtained in the core study (Hochhaus et al. 2016a). As in the core study, the rate of early molecular responses (EMR) (ratio ≤10%) at 3 months was achieved in almost all of the patients (93.3%) in this series, quite similar to the percentage found in the core study in the subgroup of patients not previously treated with imatinib (97%).

With respect to the kinetics, it is important to note that in our series, we have used GUS as control gene, which minimizes the bias introduced when *ABL1* is used as control gene in samples containing higher levels of *BCR*-*ABL1,* levels commonly found at diagnosis. In fact, the international scale is only applicable for ratios up to 10%, a range in which *BCR*-*ABL1/ABL1* is considered to reflect BCR-ABL1 levels in an almost linear way (Muller et al. 2009).

Our results show that the reduction of *BCR*-*ABL1* ^*IS*^ transcripts is biphasic, with an exponential decline of −0.05 ± 0.03 per day, which corresponds to a decline of 5% per day, in the first 3 months of therapy. The second slope starts in the third month and is roughly ten times lower. It is worth noting that in previous publications with imatinib, the second slope started approximately at 6 months, whereas for patients treated with nilotinib the turning point was at 4.8 months (Tang et al. 2011). This rate of decline is similar to that published by Michor et al. in 68 patients treated with imatinib. However, three major differences must be pointed out in our series. Firstly, they had used values at diagnosis, whereas baseline values were used in our study. Secondly, our study included four or five time point’s values between baseline and the value at 3 months. Thirdly, Michor et al. excluded patients who showed any increase in transcript during the first 12 months of therapy (Michor et al. 2005), whereas the higher number of measurements done in our series could compensate for transient increases (blips) in transcripts levels. In a previous study with a smaller group of 28 patients treated with nilotinib 400 mg twice daily, the first slope was −0.04 per day, slightly lower to that found in our study (−0.05/day) (Tang et al. 2011). However, in that study, diagnostic samples were used to calculate the slopes, and these patients were selected according to a monotonous decrement during the first year of therapy. In our series, the median HT was 11.1 days, slightly shorter than that reported in other studies with imatinib (18 days) (Huet et al. 2014). In 29 patients treated with second-generation TKI in first line, HT was similar (19 days) (Huet et al. 2014). In 52 patients treated with dasatinib in front line, median HT was not reported (Iriyama et al. 2015).

### Predictors of response

Sokal and spleen size at diagnosis retained prognostic importance. Spleen size was independently associated with MR4 at 18 months, and a larger size was strongly associated with a lower probability of MR^4^ at 18 months, reflecting the importance of intrinsic disease characteristics in the achievement of deep responses. In our study, baseline levels (i.e., immediately previous to nilotinib) were not predictive for response, even in univariate analysis, in contrast with the study done by Vigneri et al. in patients treated with imatinib, in which they found that high *BCR*-*ABL1/GUS* ratios at diagnosis were associated with lower probabilities of optimal responses (Vigneri et al. 2015). However, it is important to point out that 25 of our patients had received HU after diagnosis. Some authors have excluded patients with previous HU to measure the predictive value of the reduction of the ratio (Hanfstein et al. 2014). However, to the best of our knowledge, the possible influence of previous HU on the *BCR*-*ABL1* ^*IS*^ levels has not yet been published.

Importantly, EMR was obtained very early in our series, with 50% of the patients achieving it in the first month of therapy. Obtaining EMR at 1 month has been associated with a higher probability of complete cytogenetic response in patients treated with nilotinib as second line (Branford et al. 2010), but it has never been assessed with nilotinib as first line. In our series, the earlier time points in which the ratio had an independent predictive value on MR^4^ at 18 months was at 3 months with a cutoff value of 0.19%.

Although we have not identified a predictive ratio of MR^4^ at 18 months at a time earlier than 3 months, our results emphasize the importance of a faster kinetics. In previous studies, HT was found to discriminate two groups of patients among those who had not obtained a ratio of ≤10% at 3 months: better outcomes were seen in those who had a HT lower than 76 days (Branford et al. 2014). Likewise, an HT lower than 14 days was predictive of a cumulative higher probability of MMR by 12 months in patients treated with dasatinib as first line, although only univariate analyses were performed (Iriyama et al. 2015). In our study, an HT lower than 13 days was predictive of MR^4^ at 18 months and was independently associated with higher probability of optimal response (MMR) at 12 months.

## Conclusions

Our study showed that nilotinib in first line produced very fast responses, with 38% of the patients obtaining an MMR at 3 months. The slope of the transcript reduction is biphasic, steeper until 3 months, and the median HT is the shortest reported in the literature (11 days). A shorter HT was predictive of optimal response at 12 months and of MR^4^ at 18 months. These findings provide a rationale for assessing very early kinetics patterns in other studies with potent TKIs such as nilotinib.

## References

[CR1] Branford S, Martinelli G, Saglio G, Kim D, Shou Y, Reynolds J, Woodman RC, Kantarjian H, Hochhaus A, Radich JP (2010) Association of early molecular response to nilotinib with probability of cytogenetic response in chronic myeloid leukemia patients (pts) who fail imatinib. ASCO Meet Abstr 28:6513

[CR2] Branford S, Yeung DT, Parker WT, Roberts ND, Purins L, Braley JA, Altamura HK, Yeoman AL, Georgievski J, Jamison BA, Phillis S, Donaldson Z, Leong M, Fletcher L, Seymour JF, Grigg AP, Ross DM, Hughes TP (2014) Prognosis for patients with CML and >10% BCR-ABL1 after 3 months of imatinib depends on the rate of BCR-ABL1 decline. Blood 124(4):511–518. doi:10.1182/blood-2014-03-566323 10.1182/blood-2014-03-566323 24859364

[CR3] Corporation (2015) NP Tasigna [package insert]. Novartis Pharmaceuticals CorporationEast Hanover, NJ

[CR4] Cross NC, Melo JV, Feng L, Goldman JM (1994) An optimized multiplex polymerase chain reaction (PCR) for detection of BCR-ABL fusion mRNAs in haematological disorders. Leukemia 8:186–189 8289486

[CR5] Cross NC, White HE, Muller MC, Saglio G, Hochhaus A (2012) Standardized definitions of molecular response in chronic myeloid leukemia. Leukemia 26:2172–2175. doi:10.1038/leu.2012.104 22504141 10.1038/leu.2012.104

[CR6] Cross NC, White HE, Colomer D, Ehrencrona H, Foroni L, Gottardi E, Lange T, Lion T, Machova Polakova K, Dulucq S, Martinelli G, Oppliger Leibundgut E, Pallisgaard N, Barbany G, Sacha T, Talmaci R, Izzo B, Saglio G, Pane F, Muller MC, Hochhaus A (2015) Laboratory recommendations for scoring deep molecular responses following treatment for chronic myeloid leukemia. Leukemia 29:999–1003. doi:10.1038/leu.2015.29 25652737 10.1038/leu.2015.29 PMC4430701

[CR7] Hanfstein B, Shlyakhto V, Lauseker M, Hehlmann R, Saussele S, Dietz C, Erben P, Fabarius A, Proetel U, Schnittger S, Krause SW, Schubert J, Einsele H, Hanel M, Dengler J, Falge C, Kanz L, Neubauer A, Kneba M, Stegelmann F, Pfreundschuh M, Waller CF, Spiekermann K, Baerlocher GM, Pfirrmann M, Hasford J, Hofmann WK, Hochhaus A, Muller MC, Sakk, The German CMLSG (2014) Velocity of early BCR-ABL transcript elimination as an optimized predictor of outcome in chronic myeloid leukemia (CML) patients in chronic phase on treatment with imatinib. Leukemia 28:1988–1992. doi:10.1038/leu.2014.153 24798484 10.1038/leu.2014.153

[CR8] Hochhaus A, Rosti G, Cross NC, Steegmann JL, le Coutre P, Ossenkoppele G, Petrov L, Masszi T, Hellmann A, Griskevicius L, Wiktor-Jedrzejczak W, Rea D, Coriu D, Brummendorf TH, Porkka K, Saglio G, Gastl G, Muller MC, Schuld P, Di Matteo P, Pellegrino A, Dezzani L, Mahon FX, Baccarani M, Giles FJ (2016a) Frontline nilotinib in patients with chronic myeloid leukemia in chronic phase: results from the European ENEST1st study. Leukemia 30:57–64. doi:10.1038/leu.2015.270 26437782 10.1038/leu.2015.270 PMC4705425

[CR9] Hochhaus A, Saglio G, Hughes TP, Larson RA, Kim DW, Issaragrisil S, le Coutre PD, Etienne G, Dorlhiac-Llacer PE, Clark RE, Flinn IW, Nakamae H, Donohue B, Deng W, Dalal D, Menssen HD, Kantarjian HM (2016b) Long-term benefits and risks of frontline nilotinib vs imatinib for chronic myeloid leukemia in chronic phase: 5-year update of the randomized ENESTnd trial. Leukemia 30(5):1044–1054. doi:10.1038/leu.2016.5 10.1038/leu.2016.5 PMC4858585 26837842

[CR10] Huet S, Cony-Makhoul P, Heiblig M, Tigaud I, Gazzo S, Belhabri A, Souche D, Michallet M, Magaud JP, Hayette S, Nicolini F (2014) Major molecular response achievement in CML Patients can be predicted by BCR-ABL1/ABL1 or BCR-ABL1/GUS ratio at an earlier time point of follow-up than currently recommended. PLoS One 9:e106250. doi:10.1371/journal.pone.0106250 25203717 10.1371/journal.pone.0106250 PMC4159116

[CR11] Iriyama N, Fujisawa S, Yoshida C, Wakita H, Chiba S, Okamoto S, Kawakami K, Takezako N, Kumagai T, Inokuchi K, Ohyashiki K, Taguchi J, Yano S, Igarashi T, Kouzai Y, Morita S, Sakamoto J, Sakamaki H (2015) Shorter halving time of BCR-ABL1 transcripts is a novel predictor for achievement of molecular responses in newly diagnosed chronic-phase chronic myeloid leukemia treated with dasatinib: results of the D-first study of Kanto CML study group. Am J Hematol 90:282–287. doi:10.1002/ajh.23923 25530131 10.1002/ajh.23923

[CR12] Michor F, Hughes TP, Iwasa Y, Branford S, Shah NP, Sawyers CL, Nowak MA (2005) Dynamics of chronic myeloid leukaemia Nature 435:1267–1270. doi:10.1038/nature03669 15988530 10.1038/nature03669

[CR13] Muller MC, Cross NC, Erben P, Schenk T, Hanfstein B, Ernst T, Hehlmann R, Branford S, Saglio G, Hochhaus A (2009) Harmonization of molecular monitoring of CML therapy in Europe. Leukemia 23:1957–1963. doi:10.1038/leu.2009.168 19710700 10.1038/leu.2009.168

[CR14] Olshen A, Tang M, Cortes J, Gonen M, Hughes T, Branford S, Quintas-Cardama A, Michor F (2014) Dynamics of chronic myeloid leukemia response to dasatinib, nilotinib, and high-dose imatinib. Haematologica 99:1701–1709. doi:10.3324/haematol.2013.085977 25216683 10.3324/haematol.2013.085977 PMC4222479

[CR15] Saglio G, Kim DW, Issaragrisil S, le Coutre P, Etienne G, Lobo C, Pasquini R, Clark RE, Hochhaus A, Hughes TP, Gallagher N, Hoenekopp A, Dong M, Haque A, Larson RA, Kantarjian HM (2010) Nilotinib versus imatinib for newly diagnosed chronic myeloid leukemia. N Engl J Med 362:2251–2259. doi:10.1056/NEJMoa0912614 20525993 10.1056/NEJMoa0912614

[CR16] Tang M, Gonen M, Quintas-Cardama A, Cortes J, Kantarjian H, Field C, Hughes TP, Branford S, Michor F (2011) Dynamics of chronic myeloid leukemia response to long-term targeted therapy reveal treatment effects on leukemic stem cells. Blood 118:1622–1631. doi:10.1182/blood-2011-02-339267 21653938 10.1182/blood-2011-02-339267 PMC3156048

[CR17] Vigneri P, Stagno F, Stella S, Cupri A, Forte S, Massimino M, Antolino A, Caracciolo C, Nocilli L, Impera S, Musolino C, Turri D, Russo M, Tomaselli CAM, Rizzo M, Musso M, Morabito F, Levato L, Manzella L, Muller M, Hochhaus A, Di Raimondo F (2015) High BCR-ABL/GUSIS levels at diagnosis are associated with unfavorable responses to standard dose imatinib. Blood 126:4049

